# Impact of Online Information on Self-Isolation Intention During the COVID-19 Pandemic: Cross-Sectional Study

**DOI:** 10.2196/19128

**Published:** 2020-05-06

**Authors:** Ali Farooq, Samuli Laato, A K M Najmul Islam

**Affiliations:** 1 Department of Future Technologies University of Turku Turku Finland

**Keywords:** COVID-19, pandemic, self-isolation, behavior, protection motivation theory, cyberchondria, information overload

## Abstract

**Background:**

During the coronavirus disease (COVID-19) pandemic, governments issued movement restrictions and placed areas into quarantine to combat the spread of the disease. In addition, individuals were encouraged to adopt personal health measures such as social isolation. Information regarding the disease and recommended avoidance measures were distributed through a variety of channels including social media, news websites, and emails. Previous research suggests that the vast amount of available information can be confusing, potentially resulting in overconcern and information overload.

**Objective:**

This study investigates the impact of online information on the individual-level intention to voluntarily self-isolate during the pandemic. Using the protection-motivation theory as a framework, we propose a model outlining the effects of cyberchondria and information overload on individuals’ perceptions and motivations.

**Methods:**

To test the proposed model, we collected data with an online survey (N=225) and analyzed it using partial least square-structural equation modeling. The effects of social media and living situation were tested through multigroup analysis.

**Results:**

Cyberchondria and information overload had a significant impact on individuals’ threat and coping perceptions, and through them on self-isolation intention. Among the appraisal constructs, perceived severity (*P*=.002) and self-efficacy (*P*=.003) positively impacted self-isolation intention, while response cost (*P*<.001) affected the intention negatively. Cyberchondria (*P*=.003) and information overload (*P*=.003) indirectly affected self-isolation intention through the aforementioned perceptions. Using social media as an information source increased both cyberchondria and information overload. No differences in perceptions were found between people living alone and those living with their families.

**Conclusions:**

During COVID-19, frequent use of social media contributed to information overload and overconcern among individuals. To boost individuals’ motivation to adopt preventive measures such as self-isolation, actions should focus on lowering individuals’ perceived response costs in addition to informing them about the severity of the situation.

## Introduction

### Background

The coronavirus disease (COVID-19) pandemic is, in many ways, unique. Compared to the previous worldwide pandemic, the Spanish flu [1], the world has changed significantly. Worldwide trade, travelling, global movement, and the rate at which information is being shared over the internet have all increased drastically. Via the internet, people have access to practically an endless stream of information regarding the new emerging pandemic threat, COVID-19. Through social media, people have shared news articles as well as their own experiences about the pandemic situation, allowing instant access to the latest global developments [2].

Although the vast amount of online data can be useful for artificial intelligence and machine learning algorithms, it can be difficult for individuals to grasp and conceptualize. We envision two main problems that can emerge from excessive internet use during a worldwide pandemic such as COVID-19: (1) cyberchondria, which is defined as obsessive online searching for health-related information, typically about specific symptoms [3]; and (2) information overload, a condition in which one cannot process all the communications and informational inputs, and as a result, the information gathering process is terminated, or the whole process remains ineffective [4]. Both cyberchondria and information overload have been found to weaken human cognitive reasoning [3-5].

In this study, we investigate how these two factors, cyberchondria and information overload, impact an individual’s self-isolation intention during the COVID-19 pandemic. We use the protection motivation theory (PMT) [6] to identify intermediate constructs in between cyberchondria and information overload, and self-isolation intention. We theorized the relationships between these constructs based on previous studies (eg, [6,7]) and formulated a research model. To test the model, we used survey data from Finnish participants (N=225) and analyzed the data using partial least squares-structural equation modeling (PLS-SEM). This was followed by a post hoc analysis regarding the impact of using social media as an information source and living alone. After presenting the results, we describe the theoretical and practical implications of our findings, followed by the limitations and future work. In the end, we provide our conclusions.

### Human Behavior During Pandemics and the Case of COVID-19

In late 2019, a highly infectious new virus labelled severe acute respiratory syndrome coronavirus 2 (SARS-CoV-2) started spreading in Wuhan, China [8]. In early 2020, this viral respiratory disease had spread to most countries, and on March 11, it was declared a worldwide pandemic by the World Health Organization [9]. As of the middle of April 2020, Johns Hopkins University had reported over 2,000,000 confirmed cases of patients with COVID-19 worldwide [10]. This number is estimated to be a lot higher, as several countries are not testing cases with mild symptoms [9]. The pandemic caused governments to take action, issuing limitations on movements and meetings; closing public services, schools, and universities; and cancelling concerts and other cultural events [11,12].

There are a few established medically proven measures individuals can take during a pandemic to mitigate their chances of contracting the disease: washing hands, avoiding social contact, wearing protective masks, wearing protective gloves, and disinfecting surfaces [13]. These measures were communicated to individuals worldwide through news, social media, and other reports starting from early 2020 when the COVID-19 disease started to become a worldwide issue. In addition to individual-level health protection measures, governments issued orders to avoid large gatherings and placed areas with outbreaks in quarantine [14].

Individual-level behavior during pandemics is a result of both voluntary and government-enforced behavioral change. The benefit of government-enforced measures is that they apply to everyone and have been proven to be effective in crowd control and stopping the spread of diseases [15]. The downsides include negative impacts on the economy and citizens’ social well-being. Individuals are more motivated to comply with government-enforced measures and even adapt health measures themselves, if they understand the necessity and reasoning behind the actions [16,17]. A lack of clear communication during unusual, novel, and potentially lethal pandemic situations can lead to uncertainty and even panic among citizens [18]. Accordingly, during pandemics, intervention strategies and information bulletins aiming to propagate health information and knowledge are often used [19].

### Theoretical Foundation

One of the most used theories to explain how individuals adopt the promoted health measures, such as self-isolation, is the PMT [6]. The PMT explains individual-level behavioral responses in health-threatening situations [6]. At its core, the theory looks at motivational reasons for adopting protective measures and divides the causes into threat appraisal and coping appraisal. In the context of worldwide pandemics, threat appraisal refers to the individual’s perception of the seriousness of the situation, as well as how vulnerable they see themselves and their friends to be in the situation [7,20]. On the other hand, coping appraisal refers to the individual’s evaluation of how well they can manage in the given situation. Thus, coping appraisal can be further divided into response costs, self-efficacy, and response efficacy [7].

Previous selected work where the PMT has been used to explain human behavior during pandemics is summarized in Table 1. The literature suggests that there are significant individual differences in the likelihood of adopting health behaviors [20]. Some people feel the need to criticize or neglect suggested health behaviors [21], while others adopt them without complaint. Both threat and coping appraisals have been shown to impact protection motivation [22], with perceived severity being identified as one of the key underlying causes for both appraisals [7,20]. Protection motivation then typically leads to actual behavior [23], but there have been reports to the contrary (eg, [24]).

Despite several studies on pandemic behavior through the lens of the PMT, the existing literature has not exhaustively addressed the impact of internet sources on protection motivation and ultimately behavioral intentions. The role of the internet in pandemic situations is arguably highly complex, as it contains a myriad of information sources and social media platforms through which people can not only acquire knowledge but communicate and share experiences as well. The internet has become the primary source of information for many, but there is a large variance in the preferred online information source. Search engines and social media platforms further complicate the matter with personalized content, which can contribute to some groups of people receiving better and more accurate information regarding the pandemic situation than others. Furthermore, although studies have been done on reactions to several kinds of epidemic situations [20], COVID-19 provides a completely new context, as a pandemic of similar magnitude and impact has not been seen in modern times. To address these research gaps, we looked at cyberchondria and information overload as internet-specific constructs, and, through the lens of PMT, measured how they affect both threat and coping appraisal and, through them, the intention to self-isolate.

**Table 1:** The extant literature where PMT has been used to explain behavior during pandemics.

Author(s)	Sample	Disease	Findings
Bish and Michie [20]	Review	Multiple	Older age, being female, being non-white, and education level were associated with increased probability of adopting health behaviors. Personalized intervention strategies were suggested. Perceived threat should be emphasized as well as informing about the effectiveness of protective measures.
McNeill et al [21]	14,312 (tweets)	H1N1	People favored tweets from official sources over unverified sources. However, social media was also used to criticize and question health authorities. Social media played a role in the motivation to adopt health measures.
Miller et al [22]	84	Respiratory infections	Both threat and coping appraisal should be taken into account in interventions, and both can be used to boost protection motivation and cause behavior change.
Sharifirad et al [23]	300	H1N1	Protection motivation lead to adopting preventive behaviors. Perceived severity did not correlate with protection motivation.
Teasdale et al [7]	883	Influenza (general)	Perceived severity influenced both coping and threat appraisal. The coping appraisal was more significant than threat appraisal in determining individuals’ actions.
Williams et al [24]	230	Influenza (general)	PMT^a^ was useful for explaining intentions to engage in self-isolation behavior, but none of the PMT variables actually lead to adopting these behaviors.

^a^PMT: protection motivation theory.

### Research Model and Hypotheses

#### Cyberchondria and Perceptions

Cyberchondriac behavior is characterized by continuous impulses to go online to find further reading on a concerning health topic. Previous research has identified anxiety and a distaste for ambiguity to be predictors of cyberchondria [25] as well as exposure to too many (contradictory or unclear) information sources [26]. As such, cyberchondria can be regarded to be a product of the internet, as online sources provide a myriad of information on practically any given topic. Accordingly, at times of considerable uncertainty such as the COVID-19 pandemic, an increased amount of cases of cyberchondria can be expected to emerge.

Because the syndrome is fueled by concern for specific health issues or symptoms, it can be expected to increase the perceived severity of the given situation. Online searches for health information lead to finding more information on the topic, and humans tend to look at the worst and scariest cases first. As such, a person suffering from cyberchondria who keeps searching for more information may also experience an increased sense of vulnerability. Thus, we postulated the following hypotheses:

- Hypothesis (H)1: Cyberchondria increases perceived severity.
- H2: Cyberchondria increases perceived vulnerability.

#### The Impact of Information Overload

Due to the abundance of online and offline information regarding COVID-19, people do not have time to read and understand all available knowledge. When the amount of information crosses one’s processing capacities, information overload occurs [27]. The cognitive load theory postulates that the natural human reaction in such situations is to take a step away from the source of the information overload and retreat to a safer ground [28]. This process has successfully been used to explain a wide variety of phenomena, such as student retention in online courses [29].

The human memory is divided into two parts: long-term and working memory. New incoming information is processed by the working memory and kept as schemas in the long-term memory. The working memory can hold only a limited amount of information at a time [28]. The time it takes to process new information is dependent on existing knowledge structures and the degree that conceptual change is required to align the latest information with the existing knowledge [30]. Prior literature has reported that information overload may create fatigue [31] and reduce people’s self-regulation ability [32]. Therefore, we expect that information overload may reduce self-efficacy as well as response efficacy. It may also increase the required response costs to make a particular decision because of the feeling of uncertainty resulting from not being able to process all available information. Accordingly, we formulated the following three hypotheses:

- H3: Information overload negatively influences self-efficacy.
- H4: Information overload negatively influences response efficacy.
- H5: Information overload increases perceived response cost.

#### Factors Affecting Self-Isolation Intention

The PMT postulates that there are two aspects eventually contributing to the motivation to adopt health measures such as self-isolation: threat appraisal and coping appraisal. Building off the work of previous scholars using PMT to investigate behavior during pandemics (eg, [7]), we further divided threat appraisal into perceived severity and perceived vulnerability. Perceived severity has been shown to be perhaps the most crucial factor leading to protection motivation [20]. Thus, it should positively correlate with the self-isolation intention. Perceived vulnerability has similarly been found to increase taken health measures [33]. Accordingly, we postulated the following hypotheses:

- H6: Perceived severity increases self-isolation intention.
- H7: Perceived vulnerability increases self-isolation intention.

The other part of the PMT, coping appraisal, can be divided into self-efficacy, response efficacy, and response cost [7]. Self-efficacy refers to the individual’s beliefs in their capabilities to influence a situation. It also refers to the behavioral skills of a person [34]. On the other hand, response efficacy refers to the perception of one’s capability of being able to respond to the situation. For example, in the case of COVID-19, response efficacy consists of the ability to self-isolate at one’s own will. Response cost is the individual’s evaluation of the negative impact of specific responses. We focused primarily on the self-isolation intention, and thus, the response costs refer to what follows from self-isolation. This could mean in practice losing the opportunity to meet friends or go fishing, or even losing a job and, consequently, income. As the response costs are negative, they should have a strong negative influence on self-isolation intention. Accordingly, we postulated our final three hypotheses.

- H8: Self-efficacy positively influences the self-isolation intention.
- H9: Response efficacy negatively influences the self-isolation intention.
- H10: Response cost negatively influences the self-isolation intention.

**Figure 1:**
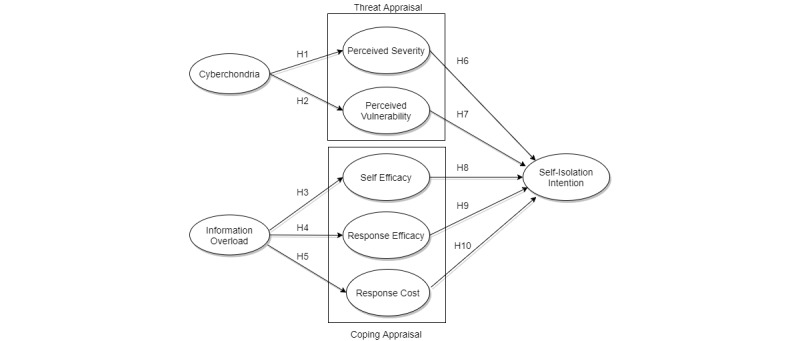
Research model explaining the relationship of cyberchondria, information overload, and perceptions and intention. H: hypothesis.

The proposed research model with the theorized hypotheses is displayed in Figure 1. The model, which is based on the PMT theory, has two root constructs connected to using the internet for information searches, cyberchondria and information overload. Cyberchondria is linked to two threat appraisal constructs, while information overload is linked to three coping appraisal constructs. All constructs from both threat and coping appraisal are linked to self-isolation intention, which is the sole dependent variable in the model and was selected to present health measures taken during the COVID-19 pandemic. Altogether the model has eight constructs and ten hypotheses.

## Methods

### Data Collection

We designed a survey by adapting validated scales from previous literature to measure the constructs. After the survey was drafted, 11 participants were asked to act as a test group to give feedback, ensuring the survey was understandable. At the beginning of the survey, the goal of the study as well as data handling procedures were clearly explained to the participants in a concise manner. Research permission was also formally asked of all participants.

An online survey tool Webropol was used for distributing the survey. The survey link was sent through email lists to students, faculty, and employees at a university in Finland. The survey received 225 responses during the time it was available from March 19-30, 2020. As all survey questions were mandatory, there were no cases of missing data. The responses were screened by the authors to ensure all responses seemed honest. More specifically, we checked if a response was random or intentionally wrong. However, no such cases were reported. Thus, all responses were deemed valid and were included in the analysis. The demographic information of the participants is summarized in Table 2.

**Table 2:** Participant’s (N=225) demographic and background information.

Factors		Distribution, n (%)
**Gender**		
	Female	147 (65.3)
	Male	73 (32.4)
	Prefer not to tell/nonbinary	5 (2.2)
**Age (years)**		
	≤25	89 (39.5)
	26-34	73 (32.4)
	35-44	34 (15.1)
	≥45	29 (12.9)
**Position in university**		
	Student	148 (65.8)
	Faculty	68 (30.2)
	Other staff	9 (4.0)
**Living situation**		
	Living alone	122 (54.2)
	Living with family/children	103 (45.8)
**Source of COVID-19^a^ information**		
	Social media	119 (52.9)
	Other channels	106 (47.1)

^a^COVID-19: coronavirus disease.

### Measures

Multi-item scales were used to measure cyberchondria, information overload, threat and coping appraisal constructs, and the dependent variable self-isolation intention. All the constructs were measured using a 5-point scale (1=strongly disagree and 5=strongly agree). A total of 25 items were used to measure eight constructs initially. Constructs involved in the study are briefly described next, and item descriptions can be seen in Table 3.

**Table 3:** Constructs, items, and reliability and validity assessments.

Construct, item		Loading	VIF^a^
**Information overload [31] (CR^b^: 0.86; AVE^c^: 0.67)**			
	“I am often distracted by the excessive amount of information on multiple channels/sources about COVID-19^d^”	0.77	1.453
	“I find that I am overwhelmed by the amount of information that I process on a daily basis from multiple channels/sources about COVID-19”	0.85	1.794
	“I receive too much information regarding the COVID-19 pandemic to form a coherent picture of what is happening”	0.82	1.481
**Cyberchondria [35] (CR: 0.82; AVE: 0.61)**			
	“After reading information about COVID-19 online, I feel confused”	—^e^	—^e^
	“I feel frightened after reading information about COVID-19 online”	0.79	1.381
	“I feel frustrated after reading information about COVID-19 online”	0.78	1.478
	“Once I start reading information about COVID-19 online, it is hard for me to stop”	0.78	1.265
**Perceived severity [36] (CR: 0.70; AVE: 0.52)**			
	“The negative impact of Coronavirus (COVID-19) is very high”	0.70	1.002
	“Coronavirus (COVID-19) can be life-threatening”	—^e^	—^e^
	“The Coronavirus (COVID-19) is a serious threat for someone like me”	0.73	1.002
**Perceived vulnerability [36] (CR: 0.81; AVE: 0.60)**			
	“I am vulnerable to contracting Coronavirus (COVID-19) in given circumstances”	0.71	1.367
	“I don't think I am likely to get the Coronavirus (COVID-19)”^f^	0.86	1.296.
	“I am at risk of catching the Coronavirus (COVID-19)”	0.74	1.567
**Self-efficacy [36] (CR: 0.84; AVE:0.64)**			
	“I am able to take avoidant measures if I want to”	0.79	1.254
	“Taking avoidant measures is difficult for me”^f^	0.84	1.652
	“Avoidant measures are easy to take”	0.78	1.617
**Response efficacy [37] (CR=0.89; AVE=0.80)**			
	“The avoidant measures are a good way of reducing the risk of contracting Coronavirus (COVID-19)”	0.90	1.614
	“The avoidant measures reduce my chance of catching the Coronavirus (COVID-19)”	0.89	1.614
**Response cost [37] (CR=0.78; AVE=0.54)**			
	“The benefits of taking avoidant measures outweigh the costs”^f^	0.72	1.146
	“I am discouraged from taking avoidant measures as they would impact my work”	0.75	1.202
	“I am discouraged from taking avoidant measures because they feel silly”	0.73	1.215
**Self-isolation intention [38] (CR=0.83; AVE=0.55)**			
	“Deliberately cancel or postpone a social event, such as meeting with friends, eating out, or going to a sports event”	0.77	1.499
	“Reduce using public transport”	0.70	1.340
	“Avoid going to shops”	0.72	1.455
	“Stay at home and study/work remotely”	0.77	1.312

^a^VIF: variance inflation factor.

^b^CR: composite reliability.

^c^AVE: average variance explained.

^d^COVID-19: coronavirus disease.

^e^Items removed due to lower loadings (<0.7).

^f^Items reverse coded for the analysis.

#### Independent Variables

Cyberchondria was measured using 4 items adapted from [35], whereas 3 items for information overload were adapted from [31].

Threat appraisal was measured using two constructs: perceived severity and perceived vulnerability. Both constructs were measured with the help of 3 items, all adapted from [36].

Coping appraisal was measured in terms of self-efficacy, response efficacy, and response cost. Self-efficacy and response cost were measured with 3 items each, whereas 2 items were used to measure response efficacy. Items for self-efficacy were adapted from [36], whereas items for both response efficacy and response cost were taken from [37].

#### Dependent Variable

The dependent variable, self-isolation intention, was measured with the help of 4 items measuring avoidance intention adapted from [38]. Before the items, we used the statement “I intend to…”.

### Data Analysis

The data was downloaded from the survey platform in .csv format, and the initial analysis was carried out in SPSS version 25 (IBM Corp). After initial screening, the data normality was checked using skewness and kurtosis. Some items had values greater than the threshold of 0.3 [39], showing the data was not normally distributed [40]. PLS-SEM has been suggested for data analysis in the case of nonnormal data.

Therefore, data were analyzed using PLS-SEM in SmartPLS 3.2 (SmartPLS GmbH) [41]. In this technique, data is analyzed in two steps. First, the measurement model is tested, ensuring reliability and validity of the constructs involved in the study. Second, an assessment of structural models is carried out, testing relationships between the constructs. As a post hoc analysis, we ran partial least squares-multigroup analysis (PLS-MGA [42] to test differences in the model due to differences in information sources and living arrangements. The post hoc analysis was supported by *t* tests (two-tailed) conducted in SPSS.

## Results

### Measurement Model Results

As mentioned, the first step in PLS-SEM analysis is to test the reliability and validity of the constructs. Reliability is assessed with internal consistency and items reliability, whereas validity consists of the convergent and discriminant validity. Internal consistency has been traditionally measured using Cronbach alpha; however, composite reliability (CR) has been recommended as a suitable measure of reliability in PLS [42]. Therefore, we considered CR for assessing internal consistency. Item reliability was assessed from the item loadings. Convergent validity was ascertained from the average variance explained (AVE), and discriminant validity was assessed with the Fornell-Larcker criterion [43]. For this purpose, we followed the accepted thresholds recommended by previous studies [41,44].

CR for all the constructs was above the threshold of 0.7 [41]. AVE for all the constructs was above 0.5. Two items, one from cyberchondria and one from perceived severity, were dropped due to item loadings below 0.7 (for details, see Table 3). In the final model, 23 items were used to measure eight constructs. The discriminant validity results based on the Fornell-Larcker criterion are shown in Table 4.

In addition, we also examined the variance inflation factor (VIF) to assess the multicollinearity. The highest VIF was 1.794 (Table 3), which was well below the threshold of 5 [45]. Thus, there was no multicollinearity issue in our data. With these assessments, we concluded that our data had a significant level of convergent and discriminant validity.

**Table 4:** Discriminant validity using Fornell-Larcker criterion.

Constructs	Self-isolation intention	Cyberchondria	Information overloading	Perceived severity	Perceived vulnerability	Response cost	Response efficacy	Self-efficacy
Self-isolation intention	0.745	—^a^	—	—	—	—	—	—
Cyberchondria	0.210	0.785	—	—	—	—	—	—
Information overloading	–0.02	0.591	0.817	—	—	—	—	—
Perceived severity	0.257	0.396	0.073	0.712	—	—	—	—
Perceived vulnerability	0.062	0.180	0.011	0.200	0.776	—	—	—
Response cost	–0.51	–0.08	0.185	–0.12	–0.01	0.738	—	—
Response efficacy	0.349	0.104	–0.04	0.115	–0.02	–0.49	0.899	—
Self-efficacy	0.396	–0.04	–0.03	–0.04	–0.18	–0.52	0.373	0.800

^a^Not available.

### Structural Model Results

**Figure 2:**
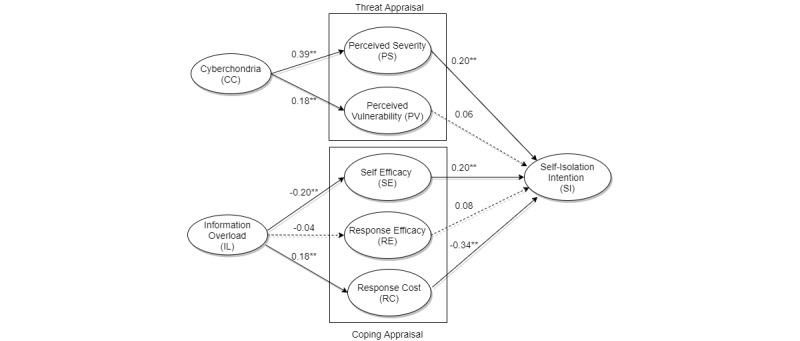
Structural model results. Significant paths are shown with solid lines, with standardized path coefficients (*P<.05, **P<.01), whereas dotted lines show insignificant relationships.

Next, we evaluated the proposed research model (see Figure 2). Complete bootstrapping with 5000 subsamples was run for the significance testing. Among the proposed relationships, 7 relationships turned significant. The model explained 34% variance in self-isolation intention.

Structural model statistics (with effect size *f*^2^) for the research model are given in Table 5. An effect size of 0.02 is considered low, 0.15 is medium, and 0.5 is large [41].

We found that both cyberchondria and information overload indirectly impacted self-isolation intention. Cyberchondria had a significant positive effect (b=0.07, *t*=2.929, *P*=.003), whereas information overload had a negative effect (b=–0.10, *t*=3.006, *P*=.003). Cyberchondria significantly impacts self-isolation intention through perceived severity, whereas information overload has an impact on it through self-efficacy and response cost.

Following the structural model analysis, we conducted several post hoc analyses to understand people’s self-isolation intentions. The results are described in the next subsections.

**Table 5:** Structural model statistics.

Hypothesis	Relationship	β	*t* test	*P* value	*f* ^2^
*H^a^1* ^b^	*CC* ^c^ *and PS* ^d^	*.39*	*7.100*	*<.001*	*0.186*
*H2*	*CC and PV* ^e^	*.19*	*2.639*	*.008*	*0.033*
*H3*	*IO* ^f^ *and SE* ^g^	–*.20*	*3.218*	*.001*	*0.044*
H4	IO and RE^h^	–.04	0.673	.50	0.002
*H5*	*IO and RC* ^i^	*.18*	*2.828*	*.005*	*0.035*
*H6*	*PS and SI* ^j^	*.20*	*3.139*	*.002*	*0.057*
H7	PV and SI	.05	0.662	.51	0.005
*H8*	*SE and SI*	*.20*	*2.998*	*.003*	*0.042*
H9	RE and SI	.07	1.067	.24	0.007
*H10*	*RC and SI*	–*0.34*	*4.374*	*<.001*	*0.112*

^a^H: hypothesis.

^b^Significant relationships are shown in italics.

^c^CC: cyberchondria.

^d^PS: perceived severity.

^e^PV: perceived vulnerability.

^f^IO: Information overload.

^g^SE: self-efficacy.

^h^RE: response efficacy.

^i^RC: response cost.

^j^SI: self-isolation intention.

### Effect of Information Source: Post Hoc Analysis

As social media is one of the major sources of information regarding the COVID-19 pandemic, we investigated if the mean values of our constructs, as well as the path coefficients, varied between the respondents who reported social media as the primary source of information and other sources (see Table 6). We observed no significant differences in the beliefs related to threat appraisal and coping appraisal of the respondents who used social media as a primary information source for COVID-19 and those who did not use social media. Similarly, no difference in self-isolation intention was found between the aforementioned groups. The level of cyberchondria and information overload was higher among respondents who used social media as a source to learn about COVID-19 in comparison to the respondents who reported using other channels.

To further see if our model (Figure 1) differs for the respondents who used social media as a source of information and those who used other channels for accumulating COVID-19 related knowledge, we ran PLS-MGA. For PLS-MGA, a *P* value of 0.05 or lower, or 0.95 or higher shows significant path differences in the groups [42].

The *R*^2^ for the self-isolation intention in the social media as an information source group was 0.40, whereas for the other channels groups, it was 0.39. As shown in Table 7, the result of PLS-MGA showed no significant differences between the two groups in most of the relationships. There were only two paths where the difference was significant. First, the effect of self-efficacy on self-isolation intention (H8) was stronger in the group that used social media as an information source compared to the other group. Second, response cost had a stronger effect on self-isolation intention (H10) in the group that used social media as an information source compared to the other group.

**Table 6:** Difference in beliefs and intention of respondents who use social media and those who use other channels to get information on the coronavirus disease.

Constructs	Social media (n=119), mean (SD)	Other channels (n=106), mean (SD)	*t* test (*df*)	*P* value
Cyberchondria	*2.96 (0.77)^a^*	*2.51 (0.80)*	*4.246 (223)*	*<.001*
Information overloading	*3.15 (0.85)*	*2.70 (0.94)*	*3.779 (223)*	*<.001*
Perceived severity	3.47 (0.63)	3.60 (0.67)	–1.485 (223)	.14
Perceived vulnerability	3.31 (0.84)	3.46 (0.77)	–1.353 (223)	.18
Self-efficacy	3.90 (0.80)	4.09 (0.58)	–1.840 (223)	.07
Response efficacy	4.40 (0.58)	4.45 (0.57)	–0.643 (223)	.52
Response cost	1.79 (0.69)	1.74 (0.59)	0.580 (223)	.56
Self-isolation intention	4.31 (0.60)	4.28 (0.61)	0.371 (223)	.71

^a^Significant differences are shown in italics.

**Table 7:** Partial least squares-multigroup analysis results for the effect of source of information (as moderator).

Hypothesis	Relationship	Social media (n=119)		Other channels (n=106)		Path difference	*P* value (social media vs other channels)
		*t* test	*P* value	*t* test	*P* value		
H^a^1	CC^b^ and PS^c^	6.707	<.001	3.854	.01	0.111	.35
H2	CC and PV^d^	1.061	.29	2.281	.02	–0.149	.34
H3	IO^e^ and SE^f^	0.679	.50	3.734	.01	0.24	.12
H4	IO and RE^g^	0.035	.97	0.839	.40	0.08	.64
H5	IO and RC^h^	1.19	.23	2.616	.009	–0.099	.58
H6	PS and SI^i^	1.561	.12	2.888	.004	–0.131	.27
H7	PV and SI	0.683	.495	1.755	.08	–0.262	.12
*H8* ^j^	*SE and SI*	*3.783*	*<.001*	*0.365*	*.72*	*.29*	*.03*
H9	RE and SI	0.429	.67	1.644	.10	–0.129	.34
*H10*	*RC and SI*	*2.843*	*.004*	*2.766*	*.006*	*0.002*	*.99*

^a^H: hypothesis.

^b^CC: cyberchondria.

^c^PS: perceived severity.

^d^PV: perceived vulnerability.

^e^IO: Information overload.

^f^SE: self-efficacy.

^g^RE: response efficacy.

^h^RC: response cost.

^i^SI: self-isolation intention.

^j^Significant relationships are shown in italics.

### Effect of Living Alone: Post Hoc Analysis

Individuals living in the same household with other people cannot generally avoid contracting diseases to one another. Large households where multiple persons reside can be seen to be at an increased risk of contracting the virus compared to single-person households. Accordingly, we also investigated if the decision making varies between the respondents who live alone compared to those living with others (see Table 8). Interestingly, we did not detect any differences in any of the constructs between the two groups.

In Table 9, we see that 33% of the variance in self-isolation intention was explained by the group living alone, whereas 37% of the variance was explained by living with others. The only path with a significant difference was between information overload and response efficacy, whereas information overload significantly affected response efficacy (negatively) among respondents who lived with others.

**Table 8:** Difference in beliefs and intention of respondents who live alone and who live with other people.

Constructs	Live alone (n=122), mean (SD)	With others (n=109), mean (SD)	*t* test (*df*)	*P* value
Cyberchondria	2.66 (0.84)	2.86 (0.78)	–1.911 (223)	.06
Information overloading	2.87 (0.93)	3.03 (0.91)	–1.321 (223)	.19
Perceived severity	3.53 (0.68)	3.54 (0.63)	–0.172 (223)	.86
Perceived vulnerability	3.43 (0.81)	3.33 (0.82)	0.918 (223)	.36
Self-efficacy	3.93 (0.73)	4.07 (0.77)	–1.416 (223)	.16
Response efficacy	4.42 (0.56)	4.44 (0.60)	–0.252 (223)	.80
Response cost	1.83 (0.65)	1.71 (0.64)	1.345 (223)	.18
Self-isolation intention	4.23 (0.61)	4.38 (0.60)	–1.863 (223)	.06

**Table 9:** Partial least squares-multigroup analysis results for the effect of living alone vs with other people (as moderator).

Hypothesis	Relationship	Live alone (n=122)		With others (n=109)		Path difference	*P* value (social media vs other channels)
		*t* test	*P* value	*t* test	*P* value		
H^a^1	CC^b^ and PS^c^	5.365	<.001	2.382	.02	0.107	.47
H2	CC and PV^d^	4.011	<.001	0.441	.66	0.245	.12
H3	IO^e^ and SE^f^	2.621	.009	2.228	.03	–0.041	.76
H4	IO and RE^g^	0.65	.52	2.379	.02	0.276	.049
H5	IO and RC^h^	2.426	.02	1.756	.08	0.012	.93
H6	PS and SI^i^	2.13	.03	1.958	.05	–0.029	.85
H7	PV and SI	1.239	.22	0.289	.77	0.136	.34
H8	SE and SI	1.442	.15	2.163	.03	–0.15	.31
H9	RE and SI	1.211	.23	0.491	.62	0.059	.67
H10	RC and SI	3.735	.01	1.826	.07	–0.071	.69

^a^H: hypothesis.

^b^CC: cyberchondria.

^c^PS: perceived severity.

^d^PV: perceived vulnerability.

^e^IO: Information overload.

^f^SE: self-efficacy.

^g^RE: response efficacy.

^h^RC: response cost.

^i^SI: self-isolation intention.

## Discussion

### Principal Results

Self-isolation intention was predicted through perceived severity (threat appraisal), as well as through self-efficacy (coping appraisal) and response cost (coping appraisal). Unlike we hypothesized, perceived vulnerability and response efficacy did not correlate with the self-isolation intention. When looking at how internet use connects to threat and coping appraisal, we noticed that cyberchondria positively affected perceived severity, whereas information overload had both negative and positive effects on coping appraisal: negative for self-efficacy and positive for response cost. Lastly, both cyberchondria and information overload had an impact on self-isolation intention via the observed intermediate constructs.

The negative impact of information overload on self-efficacy can be explained by the fact that information overload does not allow an accurate understanding of the situation at hand, and uncertainty lowers self-efficacy [46]. The positive influence of information overload on response cost can, in turn, be understood by how the uncertainty that follows from information overload makes it difficult to perceive the situation objectively. As humans have a tendency of assuming the situation to be slightly worse than what it is in reality [47], this leads to an increased perceived response cost.

We also conducted post hoc analyses to study the impacts of using social media as an information source for COVID-19 and the impact of living alone compared to living with other people. We noticed that social media users experienced a higher level of cyberchondria and information overload; however, this difference did not have a significant effect on our structural model. The above finding suggests that our model is equally suitable for explaining the impact of cyberchondria and information overload on intentions to self-isolate regardless of the information sources people use. Finally, we did not find any significant differences between people living alone compared to people living with others.

### Implications of Findings

We report three theoretical contributions from our results. First, we found that information overload distorts people’s belief system, particularly coping appraisal during a pandemic. In particular, we found that information overload negatively affected self-efficacy and positively influenced response costs. Therefore, we contribute to the prior literature discussing the outcomes of information overload [31,32] by describing how it interferes with responses during a pandemic.

Second, we observed that cyberchondria influenced the perceptions of perceived severity and perceived vulnerability. We concluded that cyberchondria affects people’s threat appraisal during pandemics such as with COVID-19. With this finding, we contribute to the literature on cyberchondria [35] by showing that it plays a significant role in motivating people to adopt recommended health measures. Although cyberchondria is generally regarded to be negative, in the case of COVID-19, it might have helped people understand the actual severity of the situation. However, it also follows from our findings that although people with cyberchondria may be early adopters of self-isolation behavior, they can, in the long run, start to suffer from stress and anxiety due to constantly seeing news and reports highlighting the severity of the situation.

Third, we extend the PMT literature on pandemics (eg, [20,21]) by employing information overload and cyberchondria as predictors of threat and coping appraisal. Prior PMT literature suggests knowledge as an antecedent of threat and coping appraisal [7]. By contrast, our study shows how negative consequences of information (cyberchondria and information overload) shape the threat and coping appraisals. We further proved in our post hoc analysis that using social media as an information source increases both cyberchondria and information overload, which may be explained by the fact that social media news is more subject to individual perceptions and lacks the objective and rigorous approach to information reporting that journalists have.

The findings also have practical implications for health behavior change system designers as well as governments, journalists, and other parties interested in impacting individual-level health behavior. The importance of internet sources and their impact on both threat and coping appraisals must be accounted for when attempting to understand human behavior during pandemics. Interventions targeted to increase an individual’s perceived severity to get them to act may unintentionally increase the cyberchondria of those who already perceive the situation to be grave, causing increased strain on people in an already unusual and stressful situation. Supplementing findings from previous studies (eg, [20]), we suggest personalized intervention strategies, where individuals suffering from cyberchondria are given reassuring and hopeful messages, and those with no intention to adopt health measures are targeted with communication that aims to increase their perceived severity of the situation.

We noticed that social media users experience greater levels of cyberchondria and information overload compared to others. The responsibility of online platforms and search engines should, thus, be brought to discussion. Social media sites and search engine developers could take measures to ensure they display clear and comprehensible information to people to avoid the negative consequences of information overload and cyberchondria while still communicating to people the severity of the pandemic and recommended health measures. However, we noted that the content in social media is at large dictated by the people who are using it. Hence, educating people on responsible and healthy social media use could help alleviate the observed negative consequences.

### Limitations and Future Work

Our work also has limitations that need to be taken into account. First, the collected data was cross-sectional and hence did not account for any change over time. For example, it is possible that information overload was experienced more heavily at the beginning of the pandemic when the novelty and uncertainty of the situation were greater. Second, participants were selected from a geographically and socially limited area. During the data collection period, Finland had fewer than 1000 confirmed cases of COVID-19, and the country also has a relatively low population density. For increased reliability, our findings could be supplemented from data collected from other countries, especially those that have been hit hard by the pandemic. Third, we chose cyberchondria and information overload as examples of internet-fueled concepts; however, other constructs could have also been used in their place or in addition to them, such as the preference of trusted sources over social media and the impact of hearing experiences about the pandemic directly from people. Finally, in our post hoc analysis, we showed that social media users experienced significantly more cyberchondria and information overload compared to others, but the content on social media was not specified. Thus, future research could look into the specific types of social media behavior and content that contribute to the observed increase in cyberchondria and information overload.

Our findings invite further research to investigate the impacts of internet-related information exposure on health behavior intentions during pandemics. The significant influence of cyberchondria on perceived severity, which further leads to the self-isolation intention, also raises ethical concerns, as a seemingly negative phenomenon (cyberchondria) may be used to motivate people to adopt recommended health measures, in this case, self-isolation. Furthermore, it is possible that continuous behavior interventions aiming to get people to self-isolate may unintentionally fuel cyberchondria for those already worried. Accordingly, behavioral intervention campaigns aiming to get the entirety of the population to self-isolate voluntarily may, after a certain threshold, begin to cause more harm than good.

Supplementing previous studies on the topic, the importance of accurate, precise, and reliable information was highlighted by our findings. The government-level quarantine and movement restrictions that were placed during the COVID-19 pandemic presented a novel research problem, which has not yet been studied, and that is how online information sources impact the adoption of recommended health behaviors during pandemics. In the case of Finland, during the time of the data collection, all participants had been ordered to work from home. This most likely translates to increased time spent on computers and online, which together with the novel and uncertain COVID-19 situation may likely have increased the amount of information read online related to the pandemic. As such, the quarantine measures may be effective at stopping the spread of the pandemic, but they may boost unhealthy internet behavior, most acutely, cyberchondria.

### Conclusions

The purpose of this study was to examine the effects of information overload and cyberchondria, two constructs measuring consequences of online information, on self-isolation intention during the COVID-19 pandemic. To understand this effect, we used the PMT framework. Accordingly, we constructed a model where we measured the impact of information overload and cyberchondria on the coping and threat appraisal construct of PMT, and their subsequent relationship with the self-isolation intention. The results revealed perceived severity and self-efficacy to positively influence self-isolation intention, while response cost had a negative effect on it. Both cyberchondria and information overload were found to be higher among those who used social media as an information source. It follows from our findings that, although cyberchondria and information overload are generally regarded to be negative, during the COVID-19 pandemic they contributed to the adoption of recommended health behavior (self-isolation). The finding that using social media as an information source increases both cyberchondria and information overload invites further research into the impact of social media on human behavior during pandemics. Finally, our findings suggest that intervention strategies motivating people to adopt health measures should focus not only on stressing the severity of the situation but also on reducing information overload via the clear structuring and communication of reliable health information. This can help mitigate cyberchondria, which we showed may arise as a side effect of a severe worldwide pandemic such as COVID-19.

## Abbreviations

AVE: average variance explained
COVID-19: coronavirus disease
CR: composite reliability
H: hypothesis
PLS-MGA: partial least squares-multigroup analysis
PLS-SEM: partial least squares-structural equation modeling
PMT: protection motivation theory
SARS-CoV-2: severe acute respiratory syndrome coronavirus 2
VIF: variance inflation factor
